# Supplementary figures and images for: 3D Spheroids of Human Primary Urine-Derived Stem Cells in the Assessment of Drug-Induced Mitochondrial Toxicity
Source: Pharmaceutics. 2022 May 11;14(5):1042. doi: 10.3390/pharmaceutics14051042 (PMC9145543; doi:10.3390/pharmaceutics14051042)

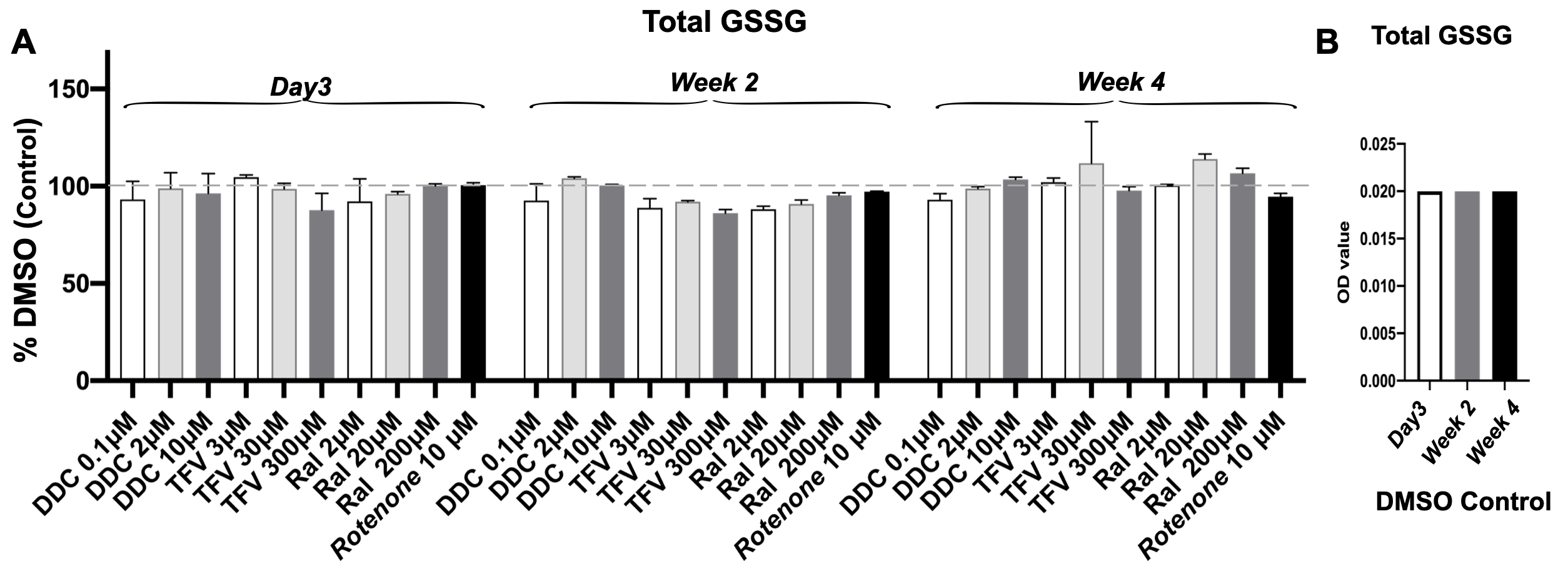

Supplement: Supplementary file 1 [file pharmaceutics-14-01042-s001.zip › supplementary files/Figure S1.png]

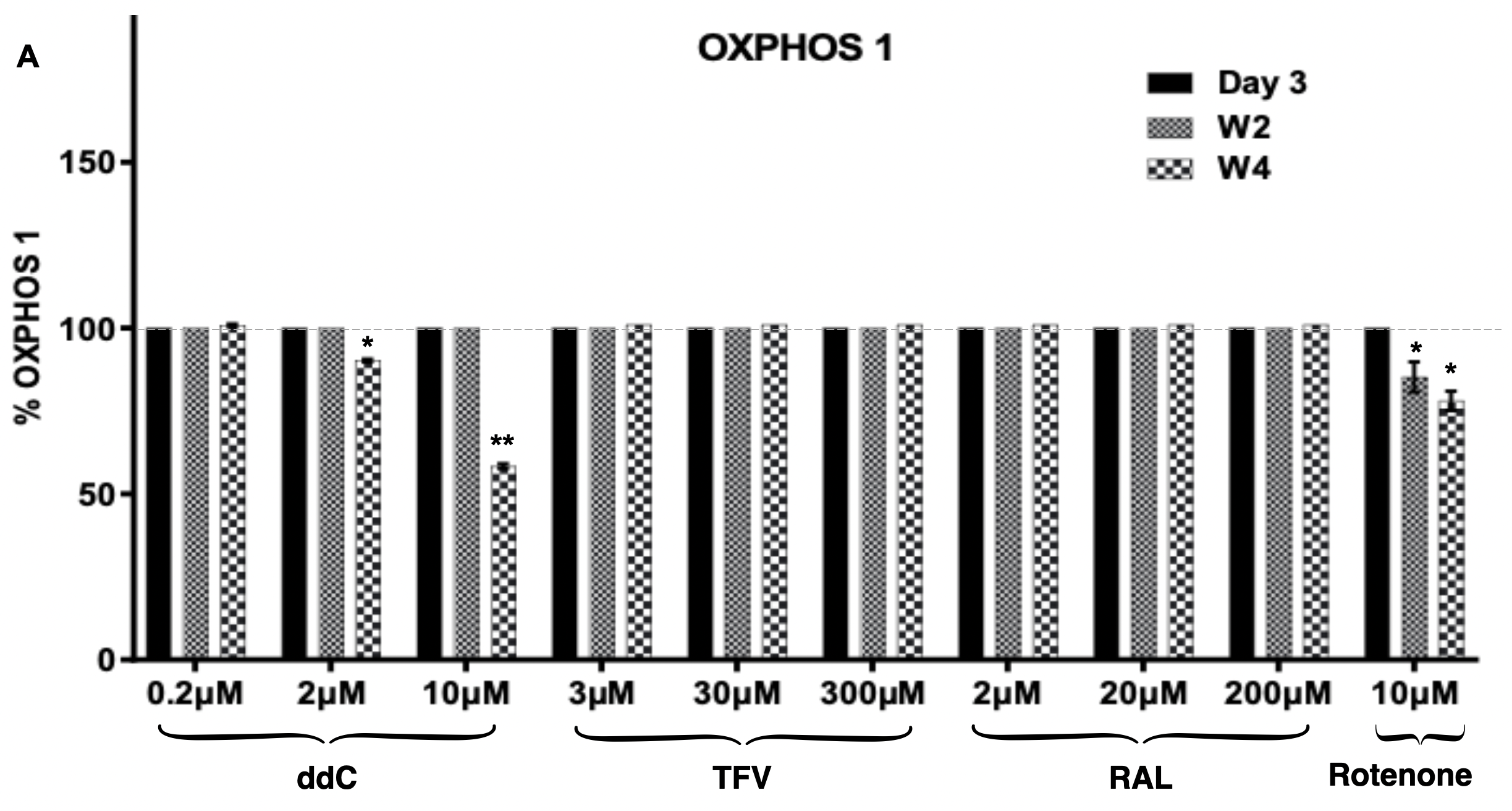

Supplement: Supplementary file 1 [file pharmaceutics-14-01042-s001.zip › supplementary files/Figure S2A.png]

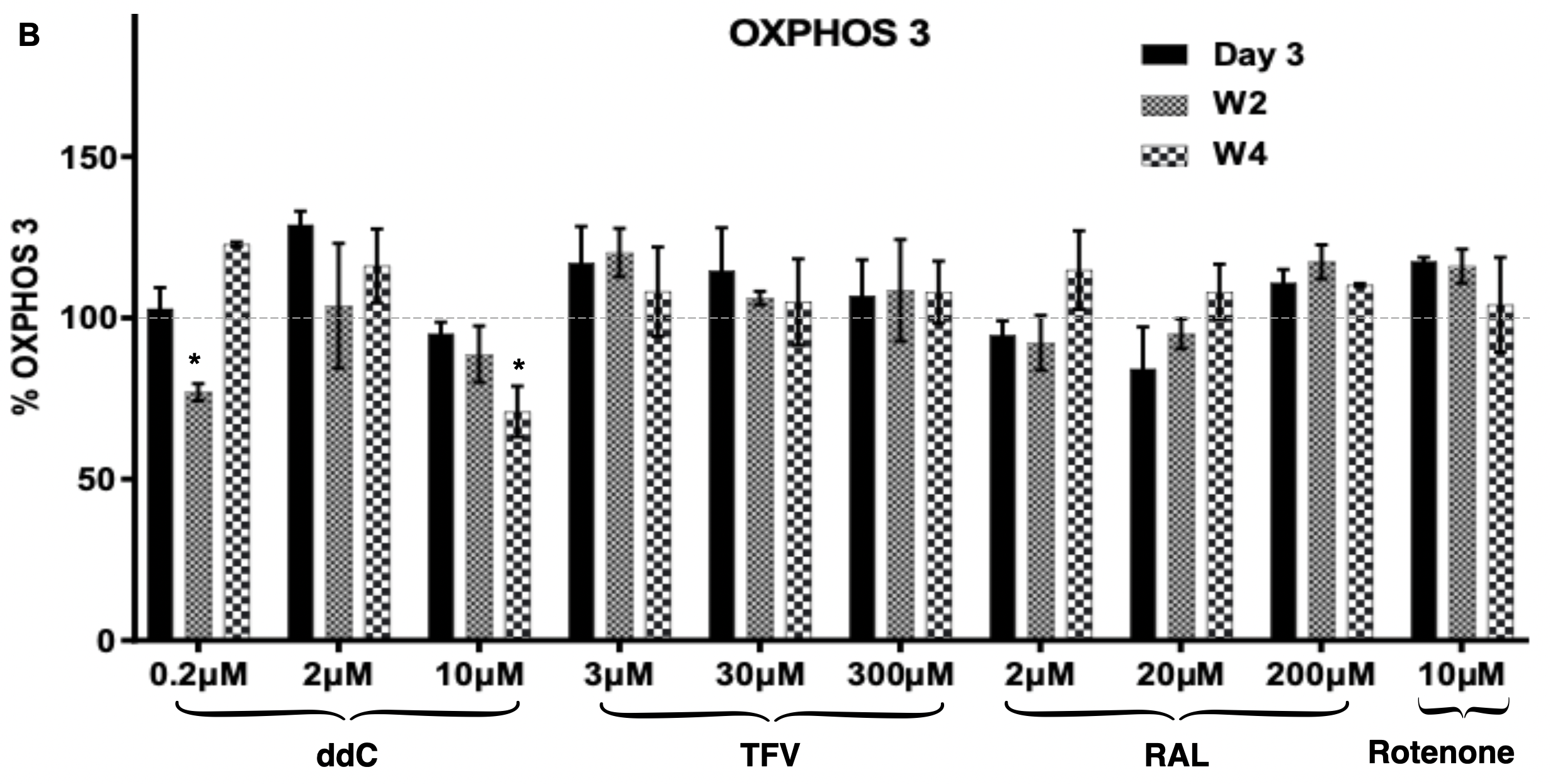

Supplement: Supplementary file 1 [file pharmaceutics-14-01042-s001.zip › supplementary files/Figure S2B.png]

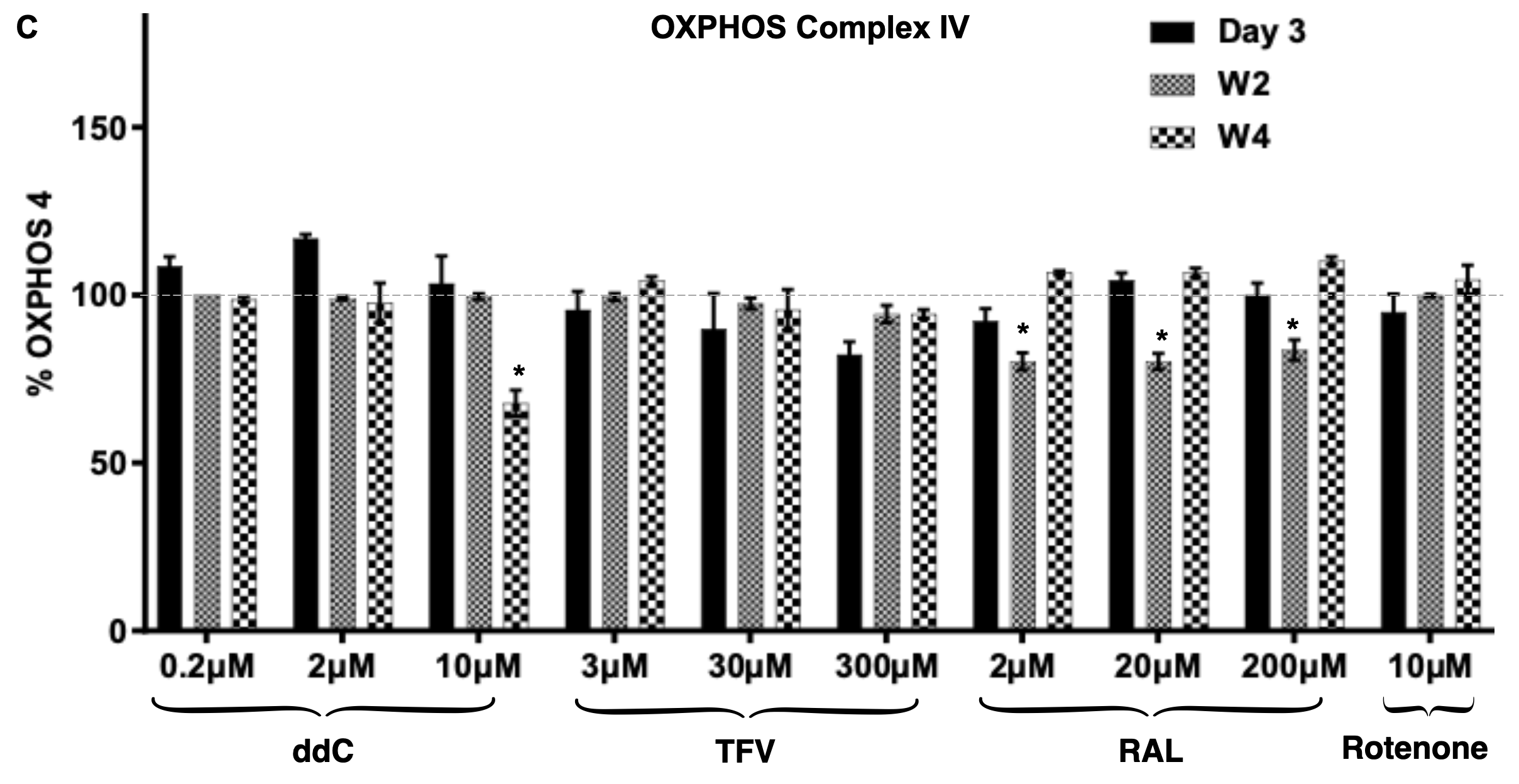

Supplement: Supplementary file 1 [file pharmaceutics-14-01042-s001.zip › supplementary files/Figure S2C.png]

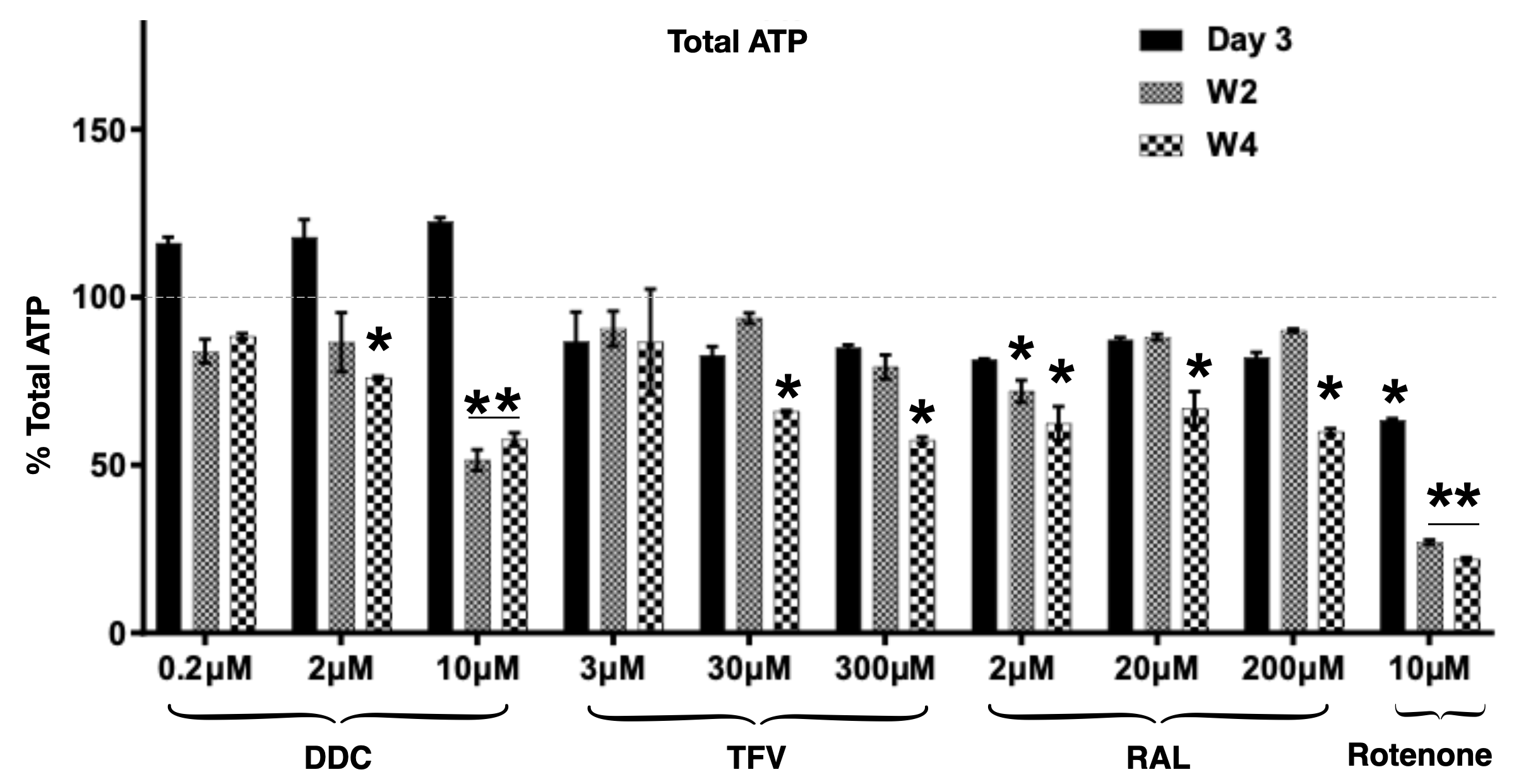

Supplement: Supplementary file 1 [file pharmaceutics-14-01042-s001.zip › supplementary files/Figure S3.png]

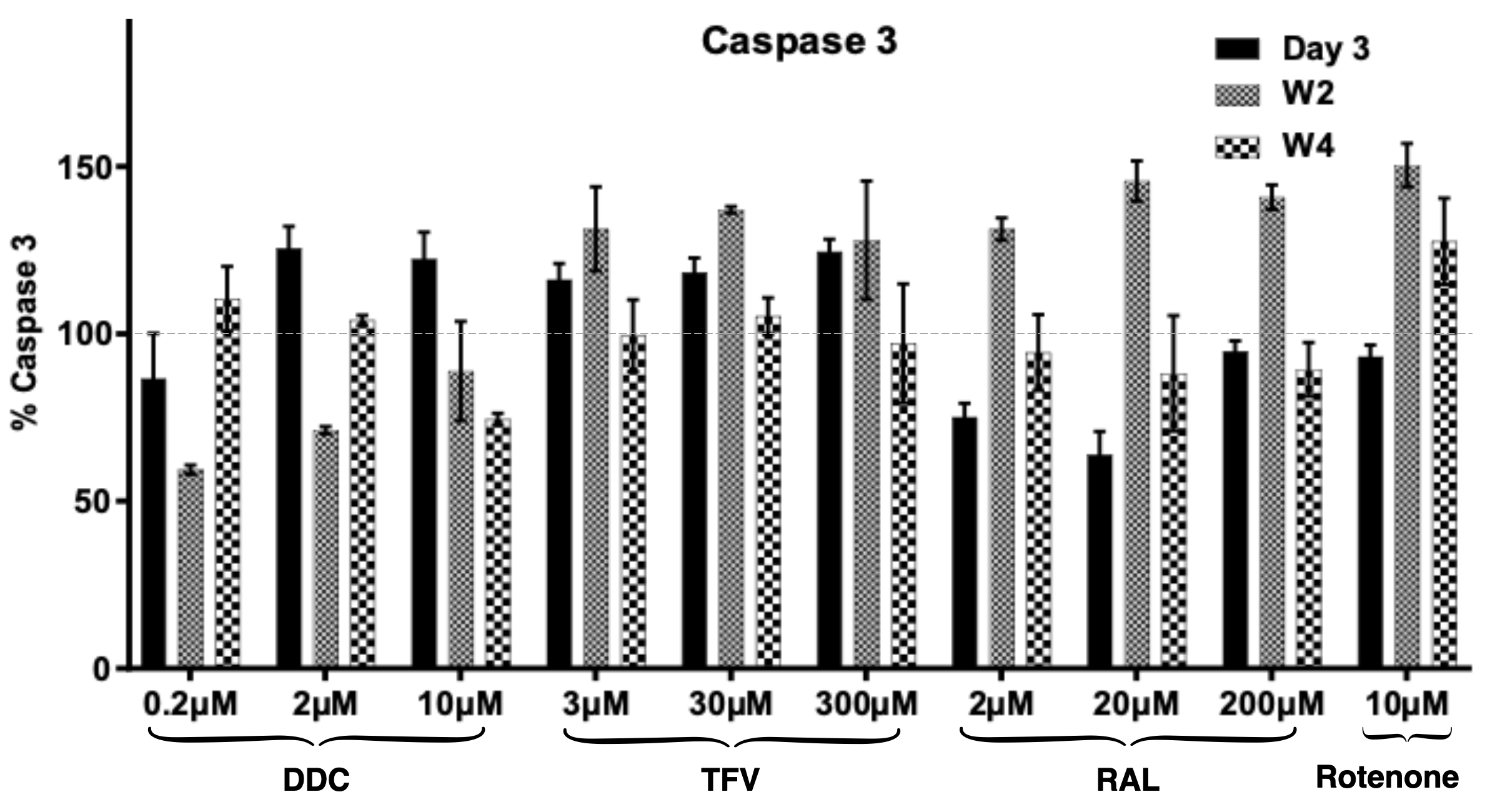

Supplement: Supplementary file 1 [file pharmaceutics-14-01042-s001.zip › supplementary files/Figure S4.png]

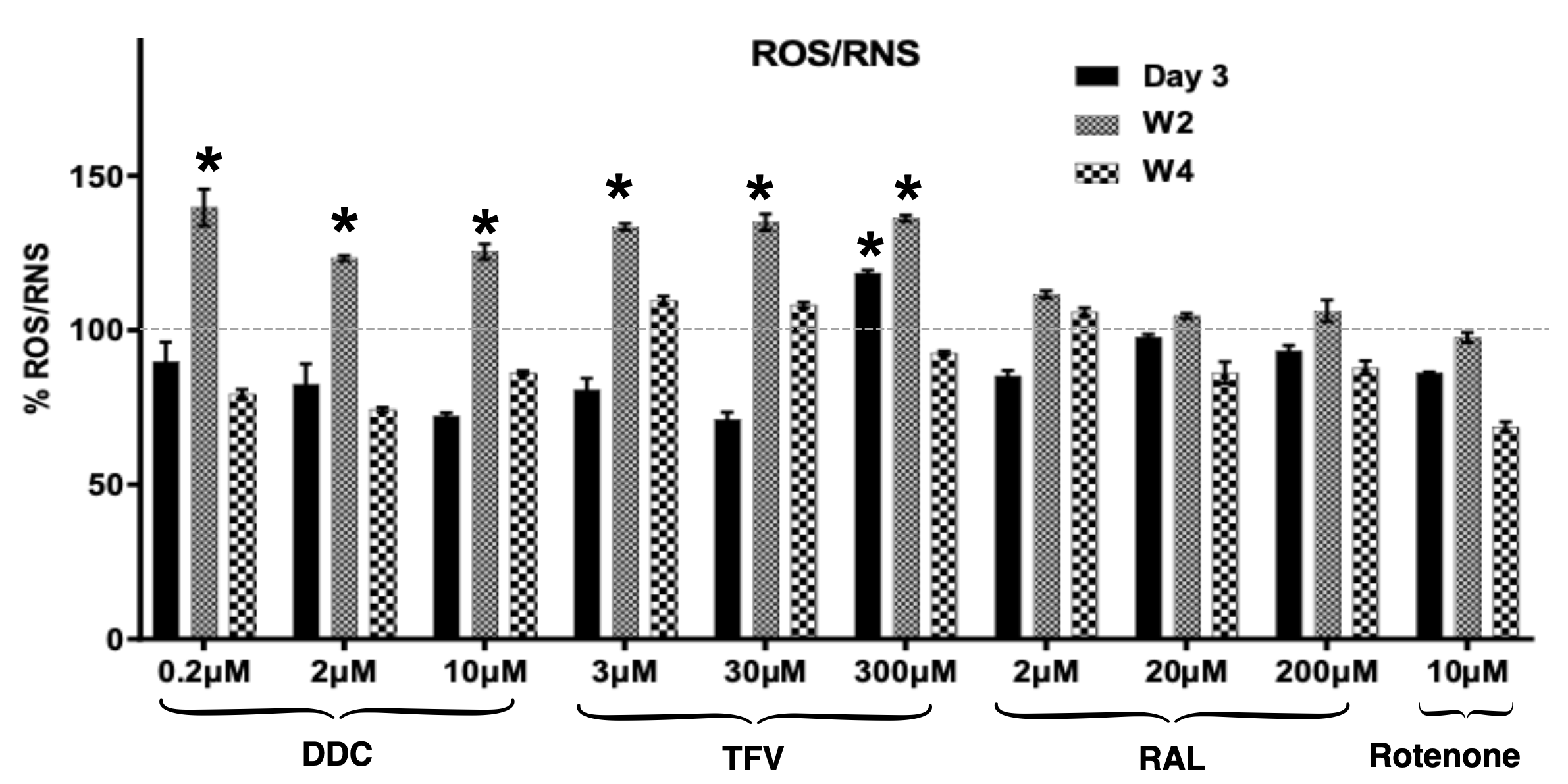

Supplement: Supplementary file 1 [file pharmaceutics-14-01042-s001.zip › supplementary files/Figure S5.png]

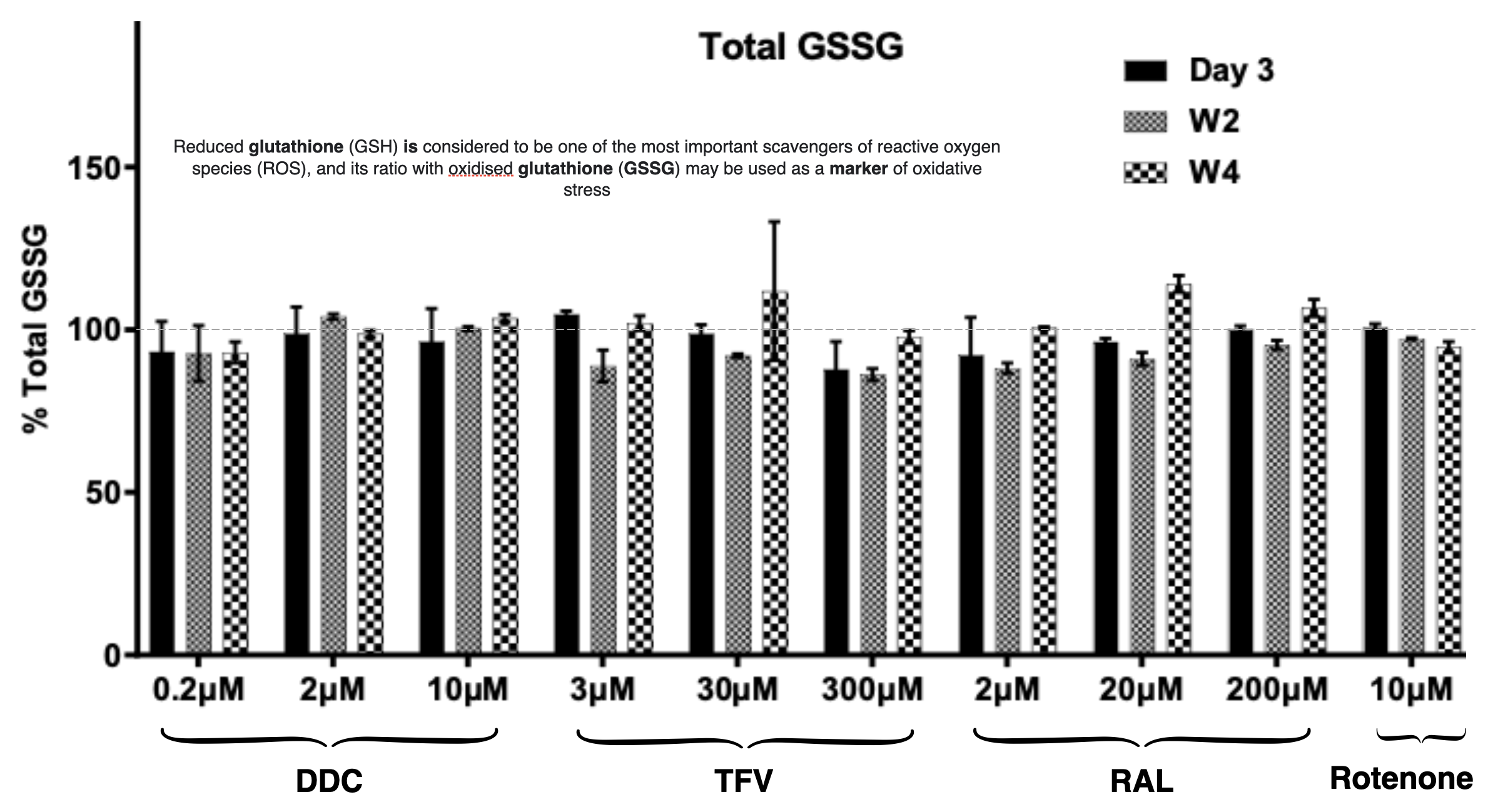

Supplement: Supplementary file 1 [file pharmaceutics-14-01042-s001.zip › supplementary files/Figure S6.png]

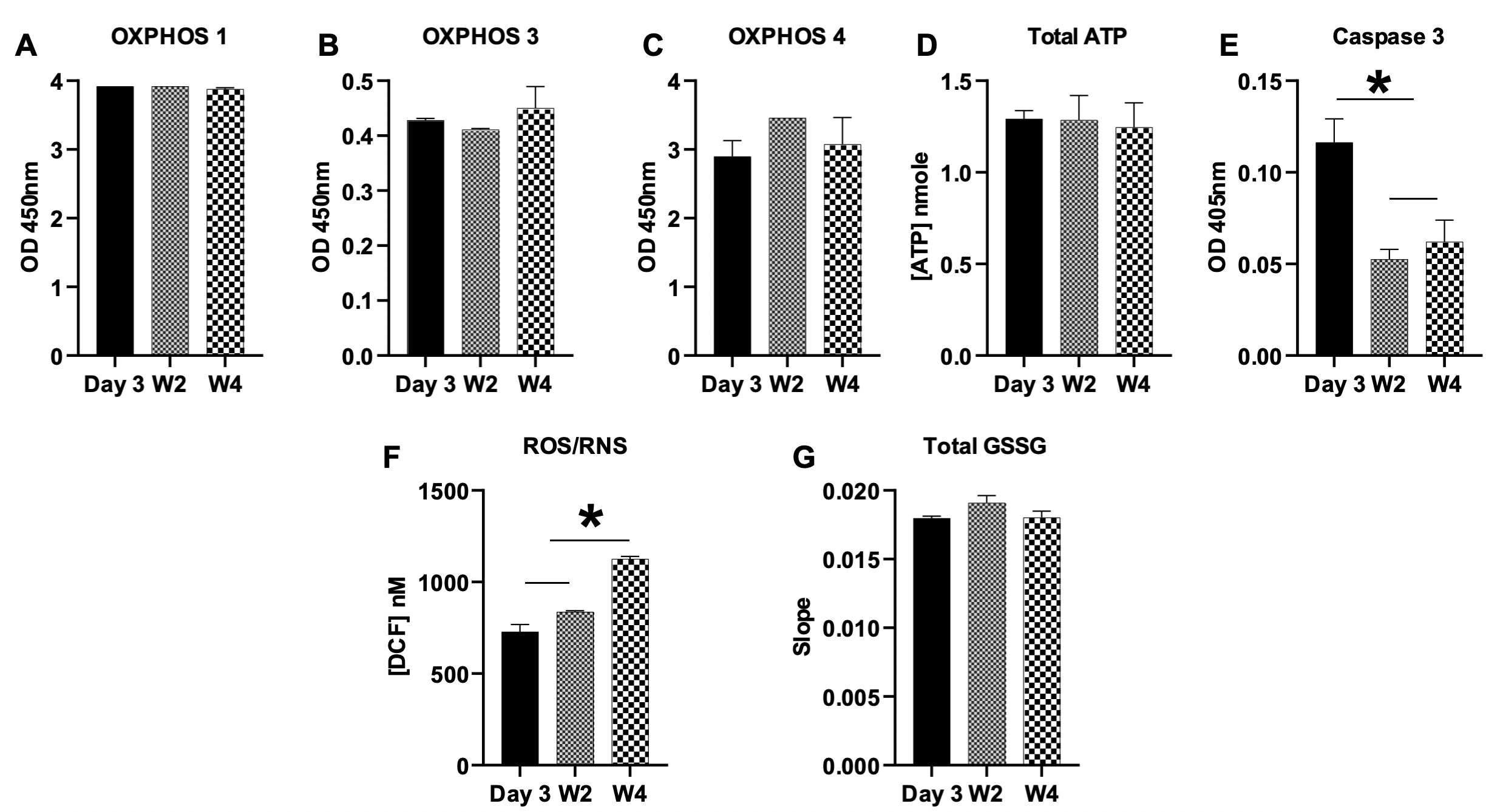

Supplement: Supplementary file 1 [file pharmaceutics-14-01042-s001.zip › supplementary files/Figure S7.png]
